# Acute effects of leg heat therapy on walking performance and cardiovascular and inflammatory responses to exercise in patients with peripheral artery disease

**DOI:** 10.14814/phy2.14650

**Published:** 2020-12-24

**Authors:** Jacob C. Monroe, Qifan Song, Michael S. Emery, Daniel M. Hirai, Raghu L. Motaganahalli, Bruno T. Roseguini

**Affiliations:** ^1^ Department of Health and Kinesiology Purdue University West Lafayette IN USA; ^2^ Department of Statistics Purdue University West Lafayette IN USA; ^3^ Department of Cardiovascular Medicine Cleveland Clinic Cleveland OH USA; ^4^ Division of Vascular Surgery Department of Surgery Indiana University School of Medicine Indianapolis IN USA

**Keywords:** blood pressure, endothelin‐1, heat therapy, intermittent claudication, peripheral artery disease

## Abstract

Lower‐extremity peripheral artery disease (PAD) is associated with increased risk of cardiovascular events and impaired exercise tolerance. We have previously reported that leg heat therapy (HT) applied using liquid‐circulating trousers perfused with warm water increases leg blood flow and reduces blood pressure (BP) and the circulating levels of endothelin‐1 (ET‐1) in patients with symptomatic PAD. In this sham‐controlled, randomized, crossover study, sixteen patients with symptomatic PAD (age 65 ± 5.7 years and ankle‐brachial index: 0.69 ± 0.1) underwent a single 90‐min session of HT or a sham treatment prior to a symptom‐limited, graded cardiopulmonary exercise test on the treadmill. The primary outcome was the peak walking time (PWT) during the exercise test. Secondary outcomes included the claudication onset time (COT), resting and exercise BP, calf muscle oxygenation, pulmonary oxygen uptake (V̇O_2_), and plasma levels of ET‐1, interleukin‐6 (IL‐6) and tumor necrosis factor‐alpha (TNF‐α). Systolic, but not diastolic BP, was significantly lower (~7 mmHg, *p* < .05) during HT when compared to the sham treatment. There was also a trend for lower SBP throughout the exercise and the recovery period following HT (*p* = .057). While COT did not differ between treatments (*p* = .77), PWT tended to increase following HT (CON: 911 ± 69 s, HT: 954 ± 77 s, *p* = .059). Post‐exercise plasma levels of ET‐1 were also lower in the HT session (CON: 2.0 ± 0.1, HT: 1.7 ± 0.1, *p* = .02). Calf muscle oxygenation, V̇O_2_, COT, IL‐6, and TNF‐α did not differ between treatments. A single session of leg HT lowers BP and post‐exercise circulating levels of ET‐1 and may enhance treadmill walking performance in symptomatic PAD patients.

## Graphical abstract

We show that a single session of leg heat therapy (HT) reduces systolic blood pressure and lowers post‐exercise circulating levels of the potent vasoconstrictor endothelin‐1. Leg HT also tended to improve peak walking time during a graded treadmill test. This practical and well‐tolerated therapy may be a useful adjunctive tool to improve cardiovascular health and restore functional capacity in PAD patients.

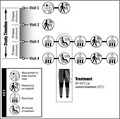

## 1 INTRODUCTION

Lower‐extremity peripheral artery disease (PAD) affects an estimated 236 million individuals worldwide (Song et al., 2019). Patients with PAD exhibit a marked reduction in exercise tolerance (Bauer et al., 2004), lower habitual physical activity levels (Sieminski & Gardner, 1997), and an accelerated functional decline when compared to their healthy counterparts (McDermott et al., 2004). A hallmark symptom of PAD is intermittent claudication (IC), defined as exertional ischemic leg pain that subsides with rest. The prevalence of IC is estimated to be <1% in those aged <50 years, increasing to 6% in those aged >65 years (Norgren et al., 2007). The genesis of the functional impairment in PAD is multifaceted and includes abnormalities in the vasculature, peripheral nerves, and skeletal muscle (Hiatt et al., 2015). Indeed, the walking impairment in PAD patients is associated, among other factors, with lowered muscle perfusion (Anderson et al., 2009; Lindner et al., 2008; Pollak et al., 2012), oxidative stress and inflammation (Gardner et al., 2018b; Nylaende et al., 2006; Pande et al., 2015), neuromuscular dysfunction (England et al., 1995; Evans et al., 2011), and numerous pathological changes in skeletal muscle, including atrophy and increased fat accumulation (McDermott et al., 2009). These patients also display abnormal responses to exercise, including an exaggerated pressor response (Kim et al., 2020a; Miller et al., 2017) and elevated circulatory levels of inflammatory mediators (Brevetti et al., 2001; Signorelli et al., 2003) and the potent vasoconstrictor endothelin‐1 (ET‐1) (Brevetti et al., 2003; Mangiafico et al., 2000). The excessive inflammatory response, and in particular the overproduction of ET‐1, is thought to antagonize muscle hyperemia during exercise, aggravate the endothelial dysfunction, and thus contribute to the development of vascular and skeletal muscle sequelae (Hiatt et al., 2015).

Emerging evidence indicates that heat therapy (HT) may be a practical therapeutic option to alleviate the symptoms of PAD and improve the quality of life of symptomatic patients. We first demonstrated that a single session of leg HT using customized, liquid‐circulating trousers perfused with warm water increases leg blood flow by ~100% and reduces blood pressure (BP) and the circulating levels of ET‐1 in symptomatic PAD patients (Neff et al., 2016). Further, we recently reported that repeated exposure to leg HT for 6 weeks improved perceived physical functioning and reduced the levels of ET‐1 by 13% when compared to a sham treatment (Monroe et al., 2020). Other HT modalities, including sauna and water bath immersion, have also been shown to elicit beneficial adaptations in PAD patients, including an improvement in walking tolerance (Akerman et al., 2019; Pellinger et al., 2019; Shinsato et al., 2010; Tei et al., 2007). In a preclinical model of PAD, repeated HT for as little as 3 days abrogated the pressor response to static muscle contraction (Qin et al., 2020).

The goal of the present study was to examine the effects of pre‐treatment with leg HT or a sham intervention on the cardiovascular responses and tolerance to a symptom‐limited exercise test on the treadmill in patients with symptomatic PAD. Building on our previous findings (Monroe et al., 2020; Neff et al., 2016), we hypothesized that a single 90‐min session of leg HT would: 1) improve calf muscle oxygenation, 2) enhance pain‐free and maximal walking time, and 3) reduce blood pressure and the levels of ET‐1 at rest and during exercise. Based upon the observations in cultured cells (Brunt et al., 2018), rodents (Chen et al., 2004), and patients with ankylosing spondylitis (Tarner et al., 2009) of reduced inflammation after exposure to heat stress, we further hypothesized that leg HT would reduce the plasma levels of interleukin 6 (IL‐6) and tumor necrosis factor alpha (TNF alpha).

## 2 METHODS

### 2.1 Subjects

Participants were identified and contacted by the Indiana Clinical and Translational Science Institute Research Network (ResNet) research assistants. After interest in participation was established, patients were contacted directly by the investigators. Additional study participants were identified by physicians from the Division of Vascular Surgery at Indiana University School of Medicine. All participants had an ankle‐brachial index (ABI) <0.90 and a history of exertional leg pain. Patients were excluded if they had uncontrolled diabetes (HbA1C >8.5, <3 months prior to taking part in the study), chronic heart failure (stage C and D), evidence of non‐healing wounds or tissue loss, recent surgical or endovascular revascularization, were HIV, HBV or HCV positive, had a body mass index (BMI) > 36, chronic kidney disease (estimated glomerular filtration rate < 30 ml/min/1.73m^2^), were receiving treatment for cancer, or if they were unable to safely and reliably complete a maximal graded treadmill test. The protocol was approved by the Institutional Review Board at Indiana University (no. 1708785351), and registered with the United States Library of Medicine on clinicaltrials.gov (NCT03435835). Written, informed consent was obtained, and all procedures adhere to the requirements of the U.S. Federal Policy for the Protection of Human Subjects (45 CFR, Part 46), and support the general ethical principles of the Declaration of Helsinki.

### 2.2 Experimental design

**Figure 1:**
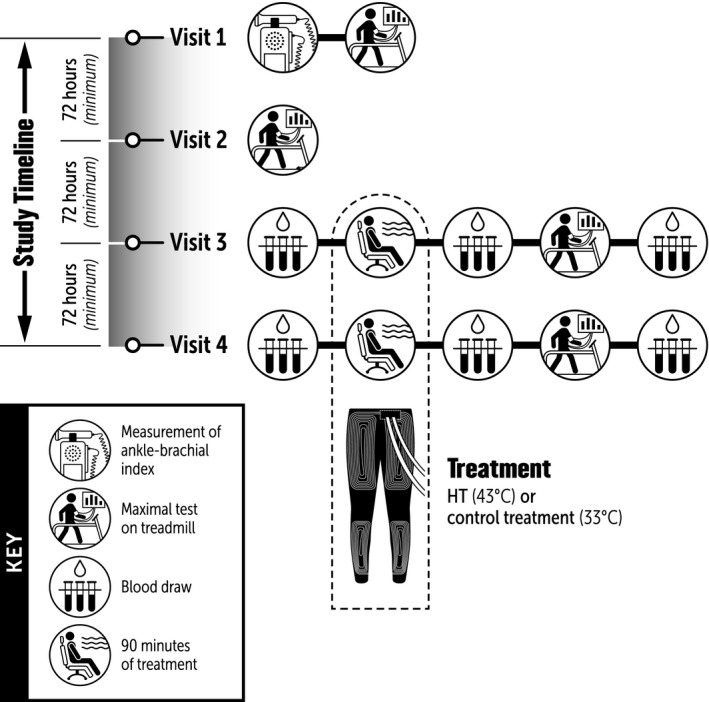
Schematic of the experimental protocol

A schematic of the experimental protocol is depicted in Figure 1. To familiarize participants with the symptom‐limited cardiopulmonary treadmill exercise test and assess the test‐retest reliability of walking tolerance, two tests were completed at baseline, at least 72 hr apart. On visit 1, participants underwent a lower extremity arterial examination to determine the ABI. Next, participants were escorted to an adjacent room and underwent resting pulmonary function testing, followed by a graded cardiopulmonary treadmill exercise test. Pulmonary oxygen uptake (V̇O_2_), and calf muscle oxygenation were measured continuously throughout the test. Similar procedures were followed on visit 2, with the exception of the lower extremity arterial exam. If the difference in peak walking time (PWT) during the exercise tests between visits 1 and 2 was greater than 20%, participants were asked to complete a third exercise test, at least 72 hr after visit 2. Once the variation in PWT was deemed acceptable (i.e., <20%), participants were assigned, using a randomized, crossover design, to undergo a single session of either HT or a control treatment (CON) prior to a symptom‐limited cardiopulmonary treadmill exercise test. The primary study outcome was the difference in PWT between HT and the CON conditions. Secondary outcomes included the differences between visits in claudication onset time (COT), blood pressure and calf muscle oxygenation at rest and during exercise, V̇O_2_ and plasma ET‐1, IL‐6 and TNF‐a concentrations post‐treatment and post‐exercise. Prior to the experimental visits, participants were asked to report to the laboratory in a fasted state (>8 hr postprandial), refrain from exercise (24 hr), smoking (>4 hr), and take their usual medications.

### 2.3 Pulmonary function tests

Pulmonary function tests were performed following the American Thoracic Society and the European Respiratory Society guidelines (Miller et al., 2005). The largest FVC and the largest FEV1 observed from all of the acceptable values are reported (Stocks & Quanjer, 1995).

### 2.4 Symptom‐limited cardiopulmonary exercise test

Exercise testing was performed in a motorized treadmill (Pro 27, Woodway, St. Paul, Minnesota, United States) following the Gardner‐Skinner protocol, which consists of walking at a constant speed (2 mph) with a 2%‐grade increase every 2 min (Gardner et al., 1991). A 12‐lead electrocardiogram (ECG) was registered continuously. Blood pressure was measured in the left arm using a stethoscope and sphygmomanometer prior and during exercise and for 10 min during recovery. Expired respiratory gases were collected breath‐by‐breath via a facemask attached to a gas analyzer (MedGraphics, CardiO2, and CPX/D system using Breeze EX Software, 142090‐001, ReVia; MGC Diagnostics, St. Paul, MN, United States). Calf muscle oxygenation was assessed using a near‐infrared spectroscopy device (NIRS; Portomon, Medis, the Netherlands), which was affixed on the skin over the medial portion of the gastrocnemius of the leg of which the participants self‐described as having the more severe pain during exercise. Participants received standardized instructions and were asked to indicate when they first began to feel leg pain with a “thumbs up” signal (defined as COT), and then give a “thumbs down” signal when they could no longer continue with the test (defined as PWT). Participants were allowed to use the handrails for balance, but were not allowed to use them as an aid for walking (i.e., pulling themselves up).

### 2.5 Heat and control treatments

Participants were instructed to ingest a core temperature sensor (CorTemp, HQ Inc., Palmetto, Florida, United States) the night before the experimental visits. Upon arrival, participants were allowed to rest for 15 min and were then instrumented with leg skin thermistors (MLT422; ADInstruments, Colorado Springs, CO, United States) and a blood pressure cuff on the upper arm that was connected to an automated monitor (SunTech CT40, SunTech Medical, Morrisville, North Carolina, United States). An intravenous catheter was placed in an antecubital vein of the left arm for blood sampling. After 30 min of rest in the semi‐recumbent position, participants were fitted with liquid‐circulating trousers as described previously (Neff et al., 2016). In the CON session, water at 33°C was circulated through the trousers for 90 min using a water pump (HTP‐1500, Adroit Medical, Louden, Tennessee, United States). We have previously demonstrated that this regimen elevates skin temperature by approximately 2°C, but does not evoke measurable changes in core body temperature, heart rate, and blood pressure (Neff et al., 2016). In the HT session, water at 43°C was circulated through the tube‐lined trousers using a heated bath circulator (HT; Aqua Relief Systems, Akron, Ohio, United States) with the goal of increasing leg skin temperature to 37°C‐38°C. The following parameters were assessed every 5 min during baseline and throughout the 90‐min intervention: intestinal temperature (HQ Inc., Palmetto, Florida, United States), leg skin temperature, blood pressure, and heart rate. Prior to the onset of the treatment and at 45 and 90 min, patients were asked to subjectively grade thermal comfort scores using an 11‐point feeling scale (Hardy & Rejeski, 1989). After the completion of the treatment, participants were promptly escorted to an adjacent room and performed a symptom‐limited exercise test as in visits 1 and 2. Blood samples were taken at baseline, at the end of the 90‐min treatment, and 10 min after the end of the exercise test.

### 2.6 Assessment of tissue oxygenation

The tissue saturation index (TSI%) of the most symptomatic leg was assessed with a commercially available NIRS system (PortaMon, Artinis Medical Systems, The Netherlands). Initially, the skin over the medial gastrocnemius of the most symptomatic leg was cleaned and, if necessary, shaved. The device was affixed using medical tape and covered with a dark cotton sleeve. The location of the monitor was traced with a permanent marker to ensure consistent placement in subsequent visits. The tracings were covered with an adhesive dressing (Tegaderm, 3M, Maplewood, MN, United States) after the exercise test and re‐drawn at each subsequent visit. Data were exported at 1Hz and the final 20‐s average of each stage was utilized for analysis.

### 2.7 Analysis of blood markers

Samples were drawn into tubes containing EDTA (BD Vacutainer, BD, Ontario, Canada). One tube was transported to the Indiana University Health Pathology Laboratory for the assessment of a routine basic metabolic panel. All other plasma samples were centrifuged at 4°C for 10 min at 1,100 g (ST16, Thermo Scientific, Waltham, MA, United States). Samples were then aliquoted and immediately placed into a −80°C freezer. Commercially available enzyme‐linked immunosorbent assay kits were used to measure the plasma concentrations of ET‐1 (DET100, Endothelin‐1 Quantikine ELISA Kit, R&D Systems, Minneapolis, MN, United States), TNF‐α (HSTA00E, Human TNF‐alpha Quantikine HS ELISA kit, R&D Systems, Minneapolis, MN, United States) and IL‐6 (HS600C, Human IL‐6 HS ELISA Kit, R&D Systems, Minneapolis, MN, United States). Blood biomarker values were corrected for potential changes in plasma volume during heating and exercise as described elsewhere (Jabbour et al., 2014).

### 2.8 Statistical analysis

All data were analyzed using SAS v9.4. Data are presented as mean ± *SD*. Variables assessed during exposure to HT or CON (blood pressure, intestinal temperature, HR, and skin temperature) were compared using a two‐way repeated‐measures analysis of variance (ANOVA). PWT and COT times were compared using a paired *t* test. All measurements taken at COT and PWT were time‐aligned (isotime) between visits 3 and 4 at the point of the earlier COT and PWT. Blood pressure during exercise was compared between groups using a two‐way repeated measures ANOVA. Missing values during the exercise test were imputed by linear interpolation, using the mean of the values from the two adjacent stages. If the value for the final stage was missing, then it was recorded as a repeat of the previous stage. TSI% and V̇O_2_ are represented using the mean of the final 20 s for each stage. Staged averages were compared using a two‐way repeated measures ANOVA. When a group‐by‐time interaction was detected, post hoc analysis compared values at each time point. A Bonferroni adjustment was applied to correct for multiple comparisons when appropriate. Statistical significance was set at *p* < .05.

## 3 RESULTS

### 3.1 Subject characteristics

Nineteen participants were consented, but three were withdrawn before receiving an experimental treatment due to the inability to safely and consistently perform the treadmill exercise test. A total of 16 participants completed all study visits. Demographic and clinical characteristics and the spirometry results are presented in Table 1. All but one of the participants was a current or past smoker. Eleven patients were taking one or more antihypertensive medications and five participants were currently taking the phosphodiesterase‐3 inhibitor cilostazol.

**Table 1:** Demographic and clinical characteristics

Age (yrs)	65.7 (5.7)
Height (cm)	177.1 (7.2)
Weight (kg)	81.6 (15.7)
ABI‐most affected leg	0.69 (0.1)
ABI‐other leg	0.92 (0.2)
BMI (kg/m^2^)	25.8 (3.8)
Systolic blood pressure (mmHg)	136.2 (13.5)
Diastolic blood pressure (mmHg)	74.7 (9.8)
FVC (L)	3.85 (0.85)
	
FEV1 (L)	2.84 (0.64)
FEV1 (%pred)	87.25 (13.45)
FEV1/FVC	73.88 (7.04)
Sex, *n* (%)	
Male	14 (87.5)
Female	2 (12.5)
Most symptomatic leg, *n* (%)	
Left	8 (50.0)
Right	8 (50.0)
Stents, *n* (%)	
No	11 (68.7)
Yes	5 (31.2)
Smoking status, *n* (%)	
Never smoked	1 (6.2)
Current smoker	9 (56.2)
Past smoker	6 (37.5)
Diabetes, *n* (%)	
No	15 (93.7)
Yes	1 (6.2)
Race, *n* (%)	
Black/African American	4 (25.0)
White	12 (75.0)
Medications, *n* (%)	
Beta Blocker	5 (31.2)
ACE Inhibitor	4 (25.0)
Statin	12 (75.0)
Cilostazol	5 (31.2)
Calcium Channel Blocker	6 (37.5)
Diuretic	3 (18.7)
Anti‐Platelet Agent	3 (18.7)
Angiotensin II Receptor Antagonist	2 (12.5)
Opioid	4 (25.0)
Other	15 (93.7)

Note

Values are as means ± *SD* or *n* (%) when indicated. ABI, ankle‐brachial index; BMI, body mass index; ACE, angiotensin‐converting enzyme; FVC, forced vital capacity; FEV1, forced expiratory volume in 1 s; FEV1 (%pred), % predicted forced expiratory volume in 1 s.

### 3.2 Reliability of exercise parameters

**Figure 2:**
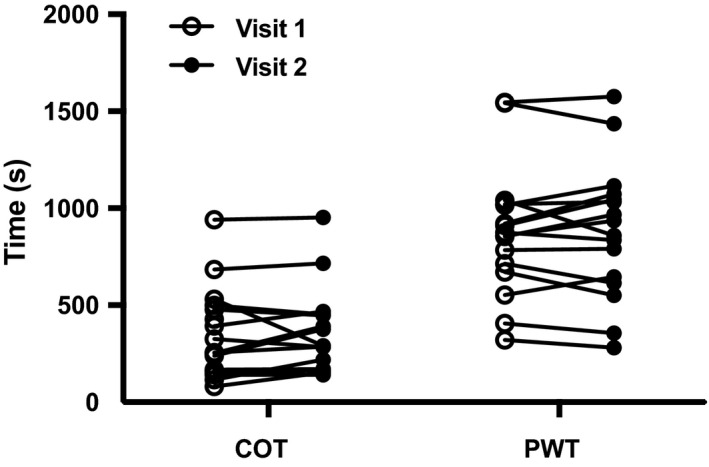
Individual values for COT and PWT at visit 1 (open circles) and visit 2 (closed circles). Data were analyzed using a paired *t* test. COT, claudication onset time; PWT, peak walking time

Four patients were asked to complete a third baseline exercise test because the difference in PWT between tests 1 and 2 was greater than 20%. After the third test, all four patients met the criteria to continue in the study. As shown in Figure 2, there were no differences between tests for COT (test 1:355 ± 232s, test 2:367 ± 224s, *p* = .49) and PWT (test 1:876 ± 334s, test 2:882 ± 348s, *p* = .84).

### 3.3 Physiological and perceptual responses to HT

**Figure 3:**
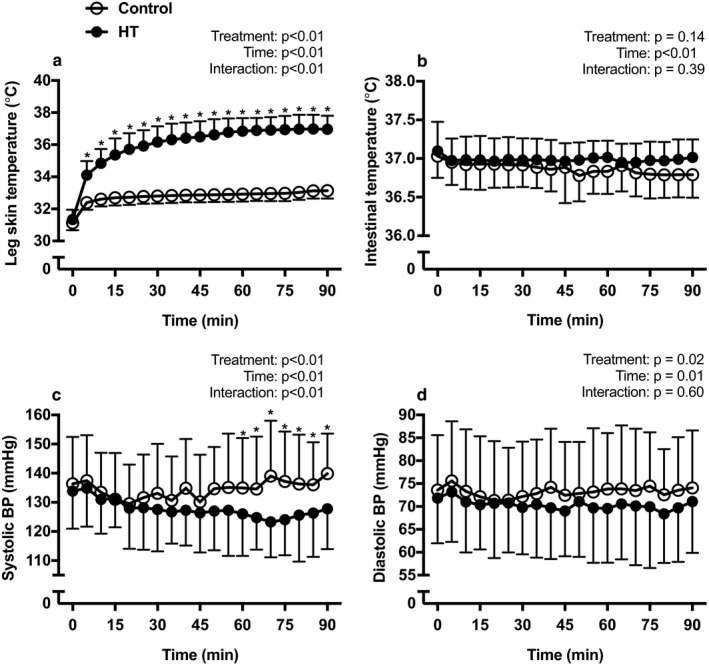
Mean leg skin temperature (panel a), intestinal temperature (panel b), systolic blood pressure (panel c), diastolic blood pressure (panel d) during exposure to 90 min of HT (closed circles) or CON (open circles). Data are presented as means ± *SD*. Data were analyzed using a two‐way repeated‐measures analysis of variance (ANOVA). *Difference between groups (*p* < .05)

The heat treatment was well‐tolerated and no adverse reactions were reported. On average, participants scored comfort levels between “Good” and “Very Good” on an 11‐point feeling scale (Hardy & Rejeski, 1989). Figure 3 depicts the average responses of leg skin temperature, intestinal temperature, and systolic (SBP) and diastolic (DBP) blood pressures during exposure to HT and CON. During HT, leg skin temperature rose from a baseline of ~ 31°C to approximately 37°C (Panel a), while intestinal temperature did not change significantly compared to CON (Panel b). SBP (Panel c), but not DPB (Panel d), was significantly reduced (*p* < .05) during exposure to HT when compared to the sham treatment. This hypotensive effect was particularly evident after approximately 60 min of exposure to the treatment (Figure 3, Panel c). HR did not differ significantly between groups during treatment.

### 3.4 Effect of HT on walking performance

**Figure 4:**
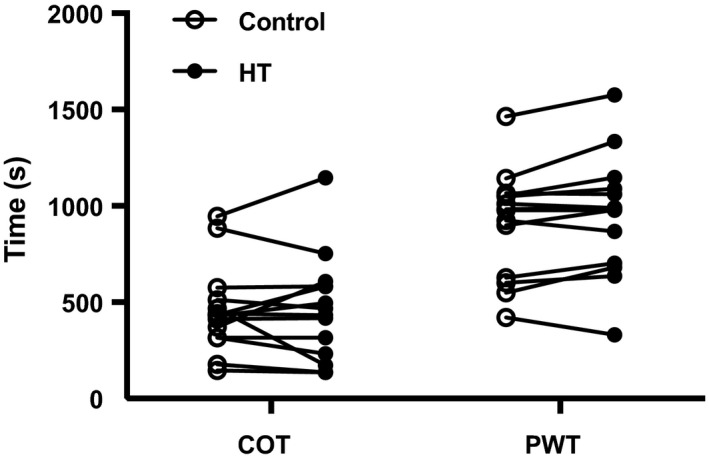
Individual values for COT and PWT after exposure to 90 min of HT (closed circles) or CON (open circles). Data were analyzed using a paired *t* test. COT, claudication onset time; PWT, peak walking time

Analysis of walking times during the symptom‐limited treadmill test after exposure to HT and CON revealed the presence of two outliers, which displayed differences in PWT between experimental visits of more than three standard deviations from the mean. One participant had a vasovagal episode due to trypanophobia after the blood draw during the HT session. On the subsequent CON visit, the patient expressed fear in experiencing another such episode and displayed a decline of 304 s in PWT. The second outlier nearly doubled the PWT detected in the baseline tests after exposure to CON, which is indicative of a lack of maximal effort during the baseline assessments. With the outliers included, there were no differences between treatments for COT (CON: 452.6 ± 53.2, HT: 442.9 ± 65.1 s, *p* = .77) and PWT (CON: 955.6 ± 84.3, HT: 947.7 ± 88.1 s, *p* = .84). After removal of the outliers, there was a trend for improved PWT in the HT group (CON: 911.9 ± 69.0, HT: 954.4 ± 77.2 s, *p* = .059) (Figure 4).

### 3.5 Blood pressure responses to exercise

**Figure 5:**
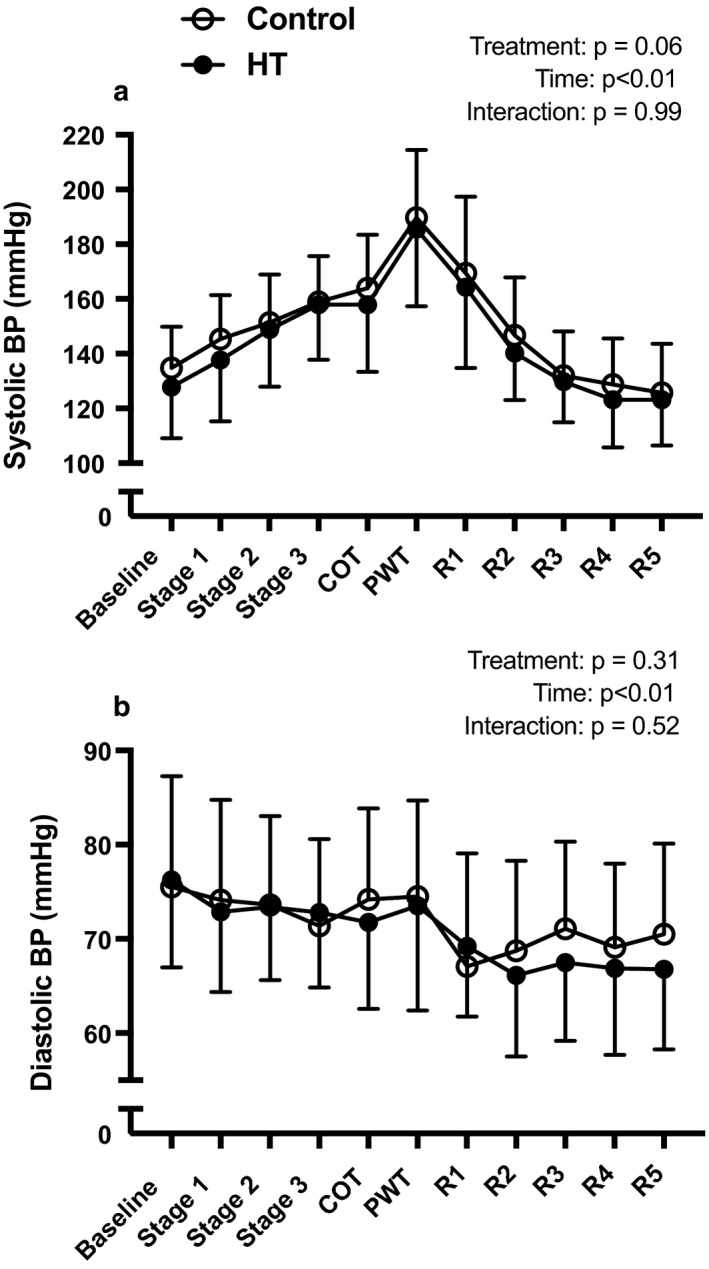
Mean systolic blood pressure (panel a) and diastolic blood pressure (panel b) during exercise after exposure to 90 min of HT (closed circles) or CON (open circles). Values are as means ± *SD*. Data were analyzed using a two‐way repeated‐measures analysis of variance (ANOVA). COT, claudication onset time; PWT, peak walking time; R1, recovery stage 1; R2, recovery stage 2; R3, recovery stage 3; R4, recovery stage 4; R5, recovery stage 5

Figure 5 depicts the changes in SBP and DBP during the graded treadmill test and during 10 min of recovery. On average, SBP was ~4 mmHg lower throughout exercise and recovery following exposure to HT when compared to the sham intervention. There was a trend for the main effect of treatment for SBP (Figure 5, Panel a; *p* = .058), but DBP was similar after HT and CON (Panel b).

### 3.6 V̇O_2_ and skeletal muscle oxygenation

**Figure 6:**
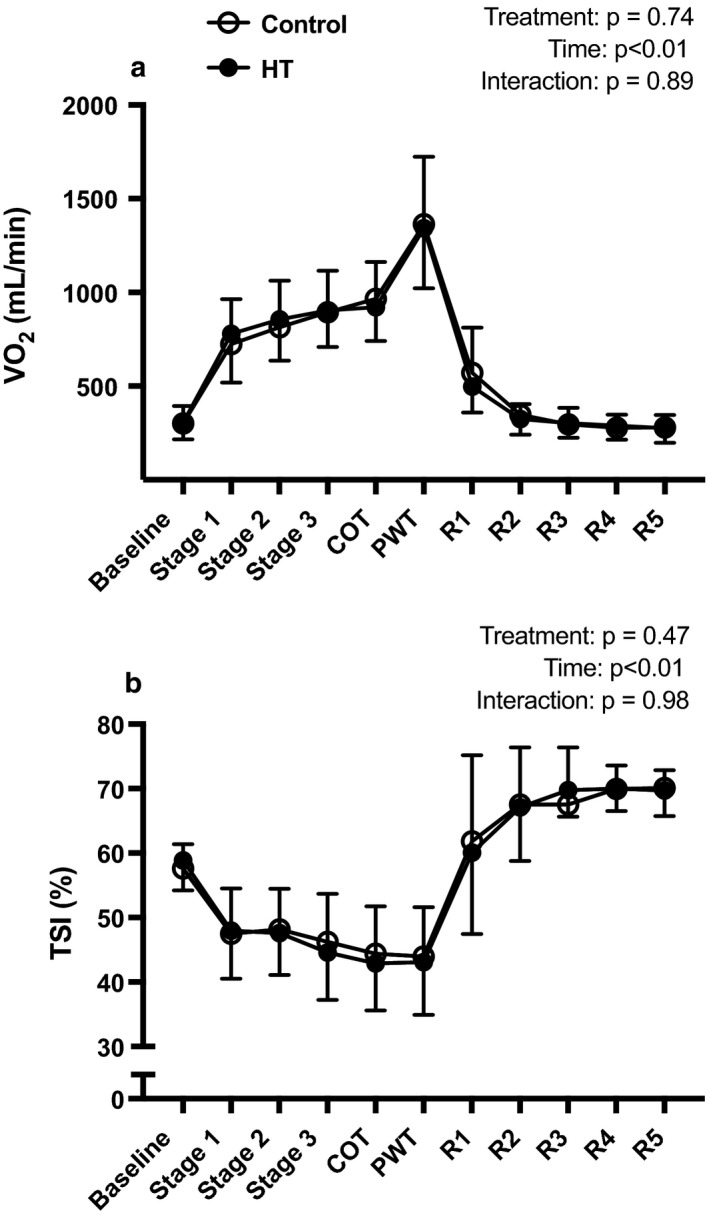
Mean responses of pulmonary V̇O_2_ (a) and TSI% (b) during exercise after exposure to 90 min of HT (closed circles, *n* = 14) or CON (open circles, *n* = 16). Data are as means ± *SD*. Data were analyzed using a two‐way repeated‐measures analysis of variance (ANOVA). HT, heat therapy; CON, control; TSI%, tissue saturation index %; R1, recovery stage 1; R2, recovery stage 2; R3, recovery stage 3; R4, recovery stage 4; R5, recovery stage 5

Figure 6 depicts the changes in V̇O_2_ (panel a) and calf muscle TSI (panel b) during the graded treadmill test and during recovery. There were no significant differences between treatments for either V̇O_2_ or TSI.

### 3.7 Plasma biomarkers

**Figure 7:**
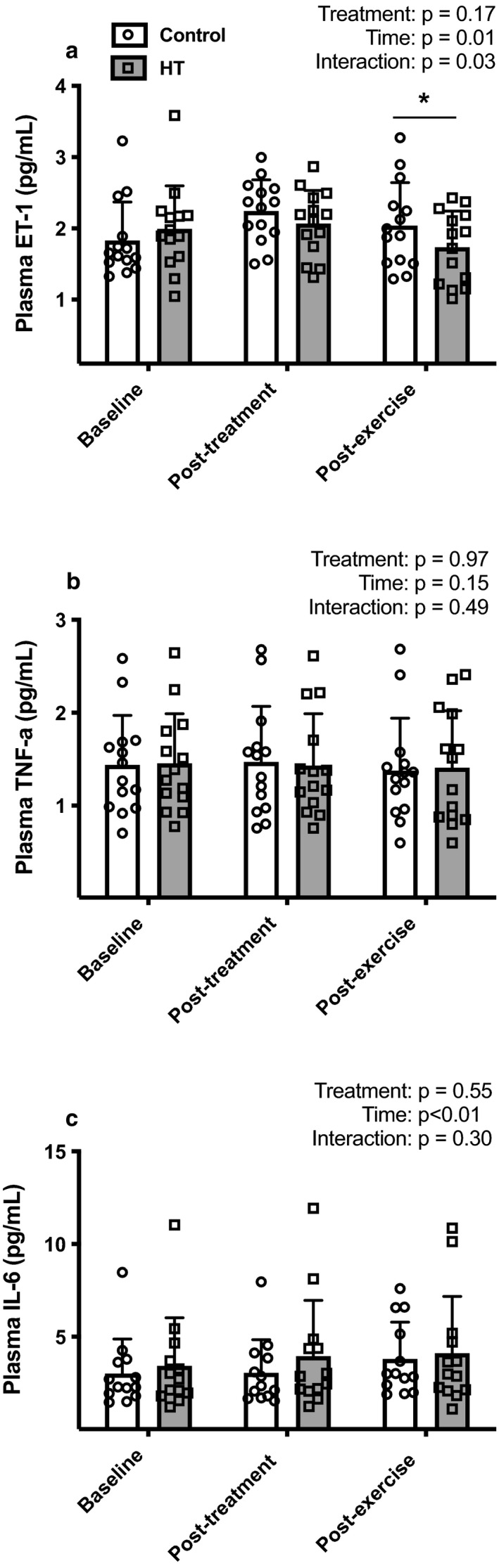
Individual and mean values of plasma ET‐1 (panel a, *n* = 14), TNF‐α (panel b, *n* = 14) and IL‐6 (panel c, *n* = 13) at rest, prior to and 10 min after exercise following HT (gray bars, open squares) or CON (white bars, open circles). Data are as means ± *SD*. Data were analyzed using a two‐way repeated‐measures analysis of variance (ANOVA). *Difference between groups (*p* < .05)

Figure 7 displays the individual and mean values for the plasma concentrations of IL‐6, ET‐1, and TNF‐α at baseline, immediately after exposure to 90‐min of HT or CON and 10 min after the maximal exercise bout. There was a significant group‐by‐time interaction for plasma ET‐1 (*p* = .03) and subsequent post hoc testing revealed a significant reduction in ET‐1 levels after exercise following HT (CON: 2.04 ± 0.16, HT: 1.73 ± 0.14 pg/mL, *p* = .02). IL‐6 levels rose significantly with exercise (*p* = .002), but to a similar degree between HT and CON. TNF‐α levels did not differ between treatments at any time point.

## 4 DISCUSSION

The primary goal of the present study was to test the hypothesis that leg heating prior to a maximal treadmill test would alleviate exertional leg pain and consequently improve walking tolerance in patients with symptomatic PAD. Based upon previous findings that leg heating enhances leg blood flow at rest (Chiesa et al., 2015; Neff et al., 2016; Thomas et al., 2017) and during exercise (Chiesa et al., 2015), we reasoned that HT would improve calf muscle oxygenation during walking, thereby delaying the onset of ischemia leg pain and extending maximal walking time in claudicants. In contrast to this hypothesis, calf muscle oxygenation during exercise and COT were unaffected by prior exposure to leg heating. However, the majority of patients experienced an increase in PWT following HT, thus revealing that a potential ergogenic effect of leg heating is unrelated to calf muscle oxygenation. We also report that HT lowered resting SBP and this hypotensive effect partially lingered throughout the exercise bout and recovery. Lastly, leg HT lowered the plasma concentration of the vasoconstrictor ET‐1 after exercise by 13% relative to baseline, while an increase of ~ 11% was detected in the control condition. Combined, these findings lend further support to the notion that HT may be a useful adjunctive therapy for symptomatic PAD (Monroe et al., 2020; Neff et al., 2016).

We previously showed that perfusion of water at 48°C through liquid‐circulating trousers elevated leg skin temperature by ~7°C and intestinal temperature by ~0.8°C and caused a profound reduction in BP in symptomatic PAD patients (Neff et al., 2016). Herein, we extend these previous observations by showing that a less strenuous thermal challenge induced by circulating water at 43°C through the trousers also causes a marked reduction in SBP. During the last 30 of the 90‐min leg HT treatment, SBP and mean arterial pressure were, on average, ~11 mmHg and ~6 mmHg lower when compared to the sham treatment. These reductions in BP occurred despite the absence of appreciable changes in intestinal temperature, thus implying that elevations in core body temperature are not a prerequisite for the hypotensive effects of HT in PAD patients. The mechanisms by which HT lowers BP remains poorly defined, but emerging evidence suggests that, in contrast to young individuals, aged adults do not display the characteristic increase in muscle sympathetic nerve activity following leg heating (Engelland et al., 2020). It has thus been proposed that a blunted neural compensatory response to HT‐induced increases in leg vascular conductance partly underlies the hypotensive effects observed in elderly individuals (Engelland et al., 2020).

The BP‐lowering effect of HT may be clinically meaningful for PAD patients, which have a heightened risk of cardiovascular morbidity and mortality (Golomb et al., 2006). Hypertension is one of the most common comorbidities and a major contributor to the elevated risk of cardiovascular events in PAD patients (Emdin et al., 2015). Although we report acute reductions in BP, it is reasonable to speculate that the cumulative effects of repeated bouts of HT would elicit a sustained reduction in BP. Along these lines, Akerman and colleagues reported that repeated spa bathing for 12 weeks reduced SBP to a greater extent than supervised exercise in PAD patients (Akerman et al., 2019). It remains to be determined, nonetheless, if the acute hypotensive effect of a single session of HT predicts the chronic BP reduction following long‐term HT treatment.

The reduction in resting BP after HT was partially sustained throughout the exercise bout and during the recovery period. This finding also holds potential clinical significance because elevated exercise BP is linked to ambulatory dysfunction (Kim et al., 2020b) and is a strong independent risk factor for all‐cause long‐term mortality in people with PAD (Liefde et al., 2008). However, it is important to note that when expressed relative to pre‐exercise levels, the increase in BP during exercise was similar following HT and CON. In other words, the modest reduction in BP during exercise after HT simply reflected the lower baseline level rather than an attenuated pressor response to exercise. These findings are incongruent with the recent observations of Qin and colleagues that repeated local leg heating for a little as 3 days abrogated the pressor response to static muscle contraction in rats subjected to femoral artery ligation (Qin et al., 2020). This discrepancy likely arises from important differences in the experimental model and protocol. First, the experiments of Qin and co‐workers were performed in young Sprague‐Dawley rats that had no exposure to the central risk factors for PAD (Qin et al., 2020). Second, the HT treatment protocol commenced only 3 days after the ligation procedure, when the natural compensation to the ischemic insult is still occurring (Ziegler et al., 2010). Third, synchronous, static muscle contractions in response to direct nerve stimulation do not mimic the mechanical, metabolic, and pressor responses to voluntary exercise.

Endothelial dysfunction, caused in part by elevated inflammation and oxidative stress, is negatively associated with walking performance and calf muscle oxygenation in patients with symptomatic PAD (Gardner et al., 2015, 2018b). Conversely, emerging evidence indicates that HT may impart resistance in endothelial cells against the deleterious effects of inflammation and oxidative stress. For instance, Brunt and colleagues recently showed that the incubation of endothelial cells with serum from individuals treated with whole‐body HT for 8 weeks reduced the cellular damage elicited by hypoxia‐oxygenation (Brunt et al., 2018). Along these lines, we reasoned that exposure to HT would reduce the circulating levels of inflammatory and vasoconstrictive agents, particularly after ischemia‐reperfusion elicited by the maximal exercise bout. We found that although HT had no measurable impact on plasma TNF‐α and IL‐6, the concentration of ET‐1 after exercise was reduced to levels below baseline in the HT condition. As ET‐1 is pro‐inflammatory and a potent vasoconstrictor (Dhaun & Webb, 2019), it is conceivable that a reduction in the post‐exercise levels of this factor following leg heating may abrogate ischemia‐reperfusion injury and possibly facilitate post‐exercise hypotension (Cucato et al., 2015), thereby augmenting the benefits of exercise in symptomatic PAD patients.

We chose to assess the effects of a single session of HT on walking performance during a graded, symptom‐limited treadmill test, as opposed to other walk tests, because of the unique possibility to record ventilatory and gas exchange parameters, BP, and calf muscle oxygenation. However, treadmill testing is associated with a significant learning effect in patients with PAD as revealed by the findings of increased PWT even in the absence of a therapeutic intervention (Hiatt et al., 2014). Patients with PAD randomized to the placebo group in drug trials also display increases in treadmill performance (Hiatt et al., 1995). To minimize this important limitation, patients in our study were asked to complete at least two baseline tests on separate days before commencing the experimental protocol. It is noteworthy that 25% of patients (4 out of16) exhibited a difference greater than 20% in PWT between the first two familiarization tests and had to complete a third test. This important finding underscores the critical importance of performing multiple baseline treadmill tests in some patients with PAD to ensure proper familiarization and consistency, and as a result, lower the potential for a placebo effect.

In a pilot study in six patients with PAD, Pellinger and colleagues observed that as little as 15 min of leg heating in a water bath heated to 42°C improved 6‐min walk distance by nearly 10% (Pellinger et al., 2019). Our findings align with these earlier observations as 9 out of 14 patients displayed an increase in PWT following HT when compared to the sham regimen. Albeit not statistically significant (*p* = .059), the average increase in PWT of ~42 s may be clinically meaningful for symptomatic PAD patients. Recent estimates of minimal clinically important differences (MCID) in walking performance by Gardner and colleagues reveal that an increase of 38 s is a small MCID after 3 months of supervised exercise training (Gardner et al., 2018a). Thus, a single session of leg HT may increase PWT as much as supervised treadmill walking, the primary treatment modality for PAD. These improvements occurred without any significant alterations in calf muscle oxygenation, which indicates that alternative mechanisms underlie the improvements in walking tolerance. One possibility is that HT‐induced analgesia (Tsuboshima et al., 2020) evoked an increase in pain tolerance, thereby enabling an increase in PWT.

### 4.1 Limitations

Several limitations of our study should also be acknowledged. Our study focused solely on patients that displayed the classical symptoms of IC, which are present in only a small fraction of the PAD population. It is estimated that ~10% of people with PAD report exertional leg pain that resolves with rest (McDermott, 2015). The vast majority of patients report either no symptoms or leg symptoms that are not consistent with IC (McDermott, 2015). Of note, asymptomatic patients have a significant functional impairment when compared to people without PAD (McDermott et al., 2001). It is thus important for future studies to examine whether the benefits of leg HT described herein extend to people with asymptomatic PAD. Another important limitation of the current study that is intrinsic to experiments examining the therapeutic value of thermal therapies is that it is not possible to blind participants to the treatment. In other words, since the thermal strain is markedly different between the sham and HT regimens, it is possible for patients to become unblinded to the treatment assignment. One possible strategy to circumvent this problem and minimize the potential positive expectancy effects in future investigations is to compare HT to an effectively administered placebo intervention, rather than a sham/control (Broatch et al., 2014; Wilson et al., 2018, 2019).

### 4.2 Summary and clinical implications

The pillars of the medical management of PAD are to reduce cardiovascular morbidity and mortality and to reduce limb morbidity (Bonaca & Creager, 2015). Our current findings, as well as other recent studies (Akerman et al., 2019; Kim et al., 2017; Monroe et al., 2020; Pellinger et al., 2019), indicate that HT may be a practical adjunctive therapy that fulfills both therapeutic goals. First, by reducing resting and exercise BP as well as the circulating levels of ET‐1, HT may improve cardiovascular health and consequently diminish the risk of MI, stroke, and death in patients with PAD. Second, by enhancing leg blood flow (Neff et al., 2016; Thomas et al., 2017) and improving muscle mass and strength (Kim et al., 2017), among other factors, HT may alleviate the leg symptoms and enhance the functional capacity of PAD patients. Third, by improving walking tolerance in some patients, leg HT may boost the adaptations to regular exercise. In mice, whole‐body heat stress augments endurance training‐induced mitochondrial adaptations (Tamura et al., 2014

). Whether similar benefits occur in people with PAD remains to be determined.

## CONFLICT OF INTEREST

No conflicts of interest, financial or otherwise, are declared by the authors.

## AUTHOR CONTRIBUTIONS

Conception and design: JCM, RLM, BTR; Data collection: JCM, MSE, RLM, BTR; Data analysis and interpretation: JCM, QS, DMH, BTR; Drafting manuscript: JCM, BTR; Critical revision and final approval of manuscript: all authors.
